# Alterations in inhibitory neuron subtype-selective transcripts in the prefrontal cortex: comparisons across schizophrenia and mood disorders

**DOI:** 10.1017/S0033291724002344

**Published:** 2024-10

**Authors:** Takeshi Okuda, Sohei Kimoto, Rika Kawabata, Yufan Bian, Makoto Tsubomoto, Kazuya Okamura, John F. Enwright, Mitsuru Kikuchi, David A. Lewis, Takanori Hashimoto

**Affiliations:** 1 Department of Psychiatry and Behavioral Science, Kanazawa University Graduate School of Medical Sciences, Kanazawa, 920-8640, Japan; 2 Department of Psychiatry, Nara Medical University School of Medicine, Kashihara, 634-8521, Japan; 3 Department of Neuropsychiatry, Wakayama Medical University School of Medicine, Wakayama, 641-8509, Japan; 4 Department of Psychiatry, University of Pittsburgh, Pittsburgh, PA, 15213, USA; 5 Research Center for Child Development, Kanazawa University, Kanazawa 920-8640, Japan; 6 Department of Neuroscience, University of Pittsburgh, Pittsburgh, PA, 15213, USA; 7 Department of Psychiatry, National Hospital Organization Hokuriku Hospital, Nanto, 939-1893, Japan

**Keywords:** bipolar disorder, major depression, postmortem, parvalbumin, somatostatin, vasoactive intestinal peptide, calretinin

## Abstract

**Background:**

In schizophrenia (SZ), impairments in cognitive functions, such as working memory, have been associated with alterations in certain types of inhibitory neurons that utilize the neurotransmitter *γ*-aminobutyric acid (GABA) in the dorsolateral prefrontal cortex (DLPFC). For example, GABA neurons that express parvalbumin (PV) or somatostatin (SST) have more prominent gene expression alterations than those that express vasoactive intestinal peptide (VIP). In bipolar disorder (BD) and major depression (MD), which exhibit similar, but less severe, cognitive impairments than SZ, alterations of transcript levels in GABA neurons have also been reported. However, the extent to which GABA neuron subtype-selective transcripts in the DLPFC are affected, and the relative magnitudes of the diagnosis-associated effects, have not been directly compared across SZ, BD, and MD in the same study.

**Methods:**

We used quantitative polymerase chain reaction to examine levels of GABA neuron subtype-selective transcripts (PV, potassium voltage-gated channel modifier subfamily-S member-3, SST, VIP, and calretinin mRNAs), as well as the pan-GABA neuron marker 67 kDa glutamate decarboxylase mRNA, in DLPFC total gray matter of 160 individuals, including those with SZ, BD, or MD and unaffected comparison (UC) individuals.

**Results:**

Relative to UC individuals, individuals with SZ exhibited large deficits in levels of all transcripts except for calretinin mRNA, whereas individuals with BD or MD showed a marked deficit only for PV or SST mRNAs, respectively.

**Conclusions:**

These findings suggest that broader and more severe alterations in DLPFC GABA neurons might contribute to the greater cognitive impairments in SZ relative to BD and MD.

## Introduction

In schizophrenia (SZ), impairments in key cognitive functions, such as working memory and executive control, have been associated with dysfunction of the dorsolateral prefrontal cortex (DLPFC) (Barch & Ceaser, 2012; Smucny, Dienel, Lewis, & Carter, 2022). This dysfunction appears to reflect, at least in part, alterations in inhibitory cortical neurons that utilize the neurotransmitter *γ*-aminobutyric acid (GABA) (de Jonge, Vinkers, Hulshoff Pol, & Marsman, 2017; Dienel & Lewis, 2019). This interpretation is based on the findings that GABA neurotransmission in the DLPFC is essential for normal working memory (Constantinidis, Williams, & Goldman-Rakic, 2002; Rao, Williams, & Goldman-Rakic, 2000; Yoon, Grandelis, & Maddock, 2016). In particular, both the parvalbumin (PV) and somatostatin (SST) subtypes of GABA neurons appear to play crucial roles in DLPFC information processing during working memory tasks (Kim et al., 2016a; Murray et al., 2015). Indeed, among the subtypes of cortical GABA neurons, PV and SST neurons appear to be prominently altered in the DLPFC of SZ individuals, as indicated by lower levels of PV and SST mRNAs (Dienel, Fish, & Lewis, 2023b; Fung et al., 2010; Hashimoto et al., 2008; Mellios et al., 2009; Morris, Hashimoto, & Lewis, 2008; Volk, Edelson, & Lewis, 2016a). Furthermore, in the DLPFC of SZ individuals, transcripts for potassium voltage-gated channel modifier subfamily-S member-3 (KCNS3), which is selectively expressed in PV neurons (Georgiev et al., 2012), and for the 67 kDa isoform of glutamate decarboxylase (GAD67), an enzyme for GABA synthesis, were shown to be lower in PV neurons (Enwright et al., 2018; Georgiev et al., 2014; Hashimoto et al., 2003), with GAD67 mRNA also suggested to be lower in SST neurons (Morris et al., 2008). In contrast, levels of vasoactive intestinal peptide (VIP) mRNA, a marker of the third major GABA neuron subtype, have been reported to be only modestly lower or unaltered in the DLPFC of SZ individuals (Fung, Fillman, Webster, & Weickert, 2014; Fung et al., 2010; Tsubomoto et al., 2019). Similarly, transcript levels for calretinin (CR), which is expressed in a subset of VIP neurons (He et al., 2016; Ma et al., 2022), were found to be unaltered in the DLPFC of SZ individuals in multiple postmortem studies (Chung et al., 2016; Fung et al., 2010; Guillozet-Bongaarts et al., 2014; Hashimoto et al., 2003; Tsubomoto et al., 2019).

Similar types of cognitive deficits, generally less severe than in SZ (Breukelaar et al., 2020; Ceylan et al., 2020; Daban et al., 2006; Reichenberg et al., 2009), have also been observed in patients with bipolar disorder (BD) or major depression (MD) throughout the course of illness, including in remission (Bortolato, Miskowiak, Kohler, Vieta, & Carvalho, 2015; Semkovska et al., 2019). Although altered levels of markers of GABA neuron subtypes have also been reported in the DLPFC of individuals with BD or MD, findings differ substantially among studies. For example, PV mRNA levels in the DLPFC were reported to be significantly lower in BD but not in MD individuals (Sibille, Morris, Kota, & Lewis, 2011), unaltered in BD individuals (Fung et al., 2014), or significantly lower in both BD and MD individuals (Chung, Chung, Bazmi, & Lewis, 2018). Similarly, SST mRNA levels were significantly lower in MD individuals but not in BD individuals (Dienel et al., 2023a; Sibille et al., 2011), whereas another study demonstrated robustly lower levels of SST mRNA in BD individuals (Fung et al., 2014).

These disparate findings may reflect differences in methods and/or statistical power across studies of mood disorders and thus it is not possible to determine from the existing literature the extent to which GABA neuron subtype-selective transcript alterations are conserved or distinct across SZ, BD, and MD. Consequently, here we simultaneously compared levels of GABA neuron subtype-selective transcripts (PV, KCNS3, SST, VIP, and CR mRNAs), as well as the pan-GABA neuron marker GAD67 mRNA, in the DLPFC from 160 individuals, including those with SZ, BD, or MD and unaffected comparison (UC) individuals (*n* = 40 in each group). The study design also enabled us to assess the degree to which observed alterations are specific to each diagnosis or associated with certain clinical features that are shared across the diagnoses (i.e. prominent mood symptoms and psychosis), or other factors that frequently cooccur across psychiatric disorders. Relative to UC individuals, individuals with SZ exhibited large deficits in levels of all transcripts except for CR mRNA, with the greatest deficits observed for transcripts in PV neurons, whereas individuals with BD or MD showed a marked deficit only for PV or SST mRNA, respectively. Neither the presence of prominent mood symptoms or psychosis nor the cooccurring factors examined appeared to have a major contribution to these alterations. These findings suggest that broader and more severe alterations of GABA neurons in the DLPFC might contribute to the greater cognitive impairments in SZ relative to BD and MD.

## Methods

### Human individuals

Brain specimens (*N* = 160) were obtained during routine autopsies conducted at the Allegheny County Office of Medical Examiner (Pittsburgh, PA) after consent was obtained from the next-of-kin. An independent team of clinicians made consensus DSM-IV diagnoses for each individual using the results of structured interviews with family members, review of medical records, and findings from neuropathology and toxicology evaluations. The same approach was used to confirm the absence of psychiatric diagnoses in the UC individuals. All procedures were approved by the University of Pittsburgh Committee for Oversight of Research and Clinical Training Involving Decedents and Institutional Review Board for Biomedical Research, as well as the Ethics Committees of Kanazawa University Graduate School of Medical Sciences and Nara Medical University.

The cohort used in this study consisted of 40 individual tetrads, with each tetrad composed of one SZ, BD, MD, and UC individual (Table 1, online Supplementary Table 1). To reduce biological variance among groups, individuals in each tetrad were matched perfectly for sex and as closely as possible for age. Individual groups did not differ in sex, age, body mass index (BMI), postmortem interval (PMI), RNA integrity number (RIN), brain pH, or tissue storage time at −80°C, but the proportion of Black individuals was significantly greater in the SZ group relative to the other groups (Table 1). SZ, BD, and MD groups did not differ in antidepressant use at time of death (ATOD), benzodiazepine and/or anticonvulsant (benzodiazepine/anticonvulsant) use ATOD, substance use disorder ATOD or death by suicide (online Supplementary Table 2). However, the proportion of individuals with antipsychotic use ATOD was greater in the SZ group (88%) than in the BD and MD groups (35% and 18%, respectively). The proportion of individuals with tobacco use ATOD was greater in the SZ group (74%) than in the MD group (45%), with the BD group intermediate (59%) (online Supplementary Table 2).

**Table 1.** Summary characteristics of individuals contributing tissue samples

Characteristic	UC, *n* = 40	SZ, *n* = 40	BD, *n* = 40	MD, *n* = 40	Statistics
Sex, Female, *n* (%)	18 (45%)	18 (45%)	18 (45%)	18 (45%)	X^2^(3) = 0.00; *p* = 1.00
Age, years, Mean (SD)	46.8 (12.8)	47.4 (12.2)	45.8 (12.4)	45.5 (11.7)	*F*_3,156_ = 0.20; *p* = 0.90
Race, Black, *n* (%)	3 (7.5%)^x^	12 (30%)^y^	1 (2.5%)^x^	2 (5.0%)^x^	X^2^(3) = 19.28; *p* < 0.01^a^
BMI, Mean (SD)	30.0 (6.3)	28.2 (7.5)^b^	29.5 (7.4)	29.2 (8.1)	*F*_3,155_ = 0.42; *p* = 0.74
PMI, hours, Mean (SD)	19.6 (5.6)	19.9 (7.4)	20.8 (7.0)	19.2 (5.8)	*F*_3,156_ = 0.46; *p* = 0.71
RIN, Mean (SD)	8.1 (0.6)	8.0 (0.6)	7.9 (0.7)	8.0 (0.6)	*F*_3,156_ = 0.77; *p* = 0.51
Brain pH, Mean (SD)	6.7 (0.2)	6.5 (0.3)	6.6 (0.3)	6.5 (0.2)	*F*_3,156_ = 1.83; *p* = 0.15
Storage Time, months, Mean (SD)	170.8 (55.2)	180.0 (63.8)	173.5 (56.4)	176.4 (47.9)	*F*_3,156_ = 0.20; *p* = 0.90

a Groups sharing the same letter do not differ from each other in post hoc tests with false discovery rate correction for multiple comparisons.

b One individual with SZ had unknown BMI.

Abbreviations: UC = Unaffected Comparison, SZ = Schizophrenia, BD = Bipolar Disorder, MD = Major Depression, PMI = postmortem interval, RIN = RNA integrity number

We also assessed whether transcript levels were influenced by a history of prominent mood symptoms in SZ or of psychosis in BD and MD. Accordingly, SZ individuals were divided into subgroups based on the absence or presence of a diagnosis of schizoaffective disorder (SZ − M and SZ + M, respectively). Similarly, based on the absence or presence of a history of psychosis, we subdivided the BD individuals (BD − P and BD + P, respectively) and MD individuals (MD − P and MD + P, respectively). These subgroups did not differ in terms of sex, age, BMI, PMI, RIN, brain pH, or tissue storage time (online Supplementary Table 3). They also did not differ in benzodiazepine/anticonvulsant use ATOD, antidepressant use ATOD, substance use disorder ATOD, or death by suicide (online Supplementary Table 4). The proportion of individuals with antipsychotic use ATOD significantly differed between MD − P and MD + P, but did not differ either between SZ − M and SZ + M or between BD − P and BD + P (online Supplementary Table 4).

### Quantitative polymerase chain reaction (qPCR)

From the fresh frozen blocks of the right hemisphere, coronal cryostat sections (40 *μ*m) were cut and total gray matter of DLPFC area 9 was dissected from the location where sections were cut perpendicular to the pial surface in a manner that minimized white matter contamination. Total RNA was isolated from gray matter homogenates in TRIzol reagent (Invitrogen, Carlsbad, CA) via RNeasy Kit (Qiagen, Valencia, CA) and converted into complementary DNA (cDNA) using SuperScript IV VILO Master Mix Kit (Thermo Fisher, Waltham, MA). The cDNA samples were used for qPCR amplification of PV, KCNS3, SST, VIP, CR, and GAD67 transcripts and three internal reference transcripts, beta-actin (ACTB), glyceraldehyde-3-phosphate dehydrogenase (GAPDH), and peptidylprolyl isomerase A (PPIA) with Power SYBR Green Master Mix and ViiA 7 Real-time PCR System (Life Technologies, Carlsbad, CA). A previous study of the same cohort demonstrated that the mean levels of these internal reference transcripts did not differ across UC, SZ, BD, and MD individuals (Chung et al., 2018). All primer sets used in qPCR were previously confirmed to yield an amplification efficiency >98% and single specific products of the predicted sizes (online Supplementary Table 5) (Tsubomoto et al., 2019; Volk et al., 2016a). Samples from all individuals in each tetrad were processed together throughout all experimental stages. Specifically, for all individuals in each tetrad, all nine transcripts were amplified as quadruplicates on the same 384-well plate. For each transcript, cycle threshold (CT) was determined as an average across quadruplicates. The difference in CT (dCT) for each target transcript of interest was calculated by subtracting the mean CT of ACTB, GAPDH, and PPIA mRNAs from the mean CT of the target transcript. Because − dCT represents the log_2_-transformed expression ratio of each target transcript to the reference transcripts, the expression level of the target transcript (relative to the geometric mean of internal reference transcripts) was determined as 2^−dCT^ (Vandesompele et al., 2002).

### Statistics

Analyses were implemented in R version 4.2.2. Linear models were applied to compare transcript levels across individual groups with transcript level as the dependent variable; diagnosis as a fixed effect; and sex, age, PMI, RIN, brain pH, and tissue storage time as covariates. *F*-tests were computed from linear models to assess the overall effect of diagnosis, followed by post hoc comparisons across the four individual groups using Tukey's honestly significant difference test, with the significance level set at 0.05. To compare the magnitude of transcript alterations in each diagnostic group, Cohen's *d* (Cohen, 1988) was calculated relative to UC individuals for each transcript, with 95% confidence intervals (CI) as described previously (Nakagawa & Cuthill, 2007). The magnitude of the diagnosis effect was interpreted based on the following criteria: |Cohen's *d*| = 0.2–0.4, small effect; |Cohen's *d*| = 0.4–0.6, moderate effect; |Cohen's *d*| ⩾ 0.6, large effect (Cohen, 1988). Negative Cohen's *d* values indicate that mean transcript levels are lower in each diagnostic group relative to the UC group.

We also compared transcript levels between subgroups defined by the history of prominent mood symptoms or psychosis in each diagnostic group (i.e. SZ − M v. SZ + M, BD − P v. BD + P, and MD − P v. MD + P). *F*-tests were performed with the history of prominent mood symptoms or psychosis as the main effect and sex, age, PMI, RIN, brain pH, and tissue storage time as covariates.

To assess the potential effects of cooccurring factors that have been frequently associated with these psychiatric diagnoses, such as the use of prescription drugs (antidepressant, antipsychotic, and benzodiazepine/anticonvulsant) ATOD, substance use disorder ATOD, death by suicide and tobacco use ATOD, we focused on PV, KCNS3, SST, VIP, and GAD67 mRNAs that exhibited statistically significant alterations in at least one of the three diagnostic groups relative to the UC group. Transcript levels were compared between individuals with and without each of the cooccurring factors within the relevant diagnostic group(s). *F*-tests were performed with each cooccurring factor as the main effect and diagnosis, sex, age, PMI, RIN, brain pH, and tissue storage time as covariates. Holm's procedure was used to correct for multiple comparisons across the six cooccurring factors (Holm, 1979).

## Results

### Effect of psychiatric diagnosis on transcript levels

**Figure 1.**
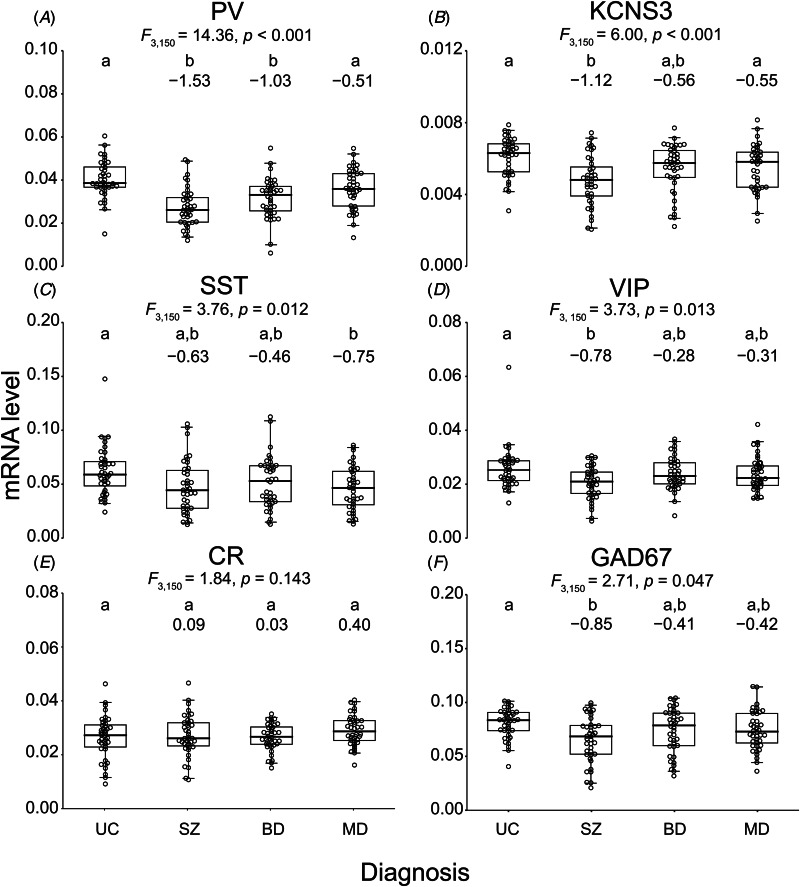
Transcript levels relative to internal reference transcripts across unaffected comparison (UC), schizophrenia (SZ), bipolar disorder (BD), and major depression (MD) individuals. In each panel (A–F), the results of *F*-tests indicate the overall diagnosis effect for parvalbumin (PV) (A), potassium voltage-gated channel modifier subfamily-S member-3 (KCNS3) (B), somatostatin (SST) (C), VIP (D), calretinin (CR) (E), and 67 kDa glutamate decarboxylase (GAD67) (F) mRNAs. Individual groups not sharing the same letter are significantly different by post hoc Tukey's test with the *α* level set at 0.05. Shown below the letters are Cohen's *d* effect sizes relative to UC individuals. Circles indicate transcript levels of individuals in UC, SZ, BD, and MD groups. Box plots depict the median, and 25th and 75th percentiles, with whiskers extending to the 95th percentiles of each distribution.

The effect of diagnosis was significant for all transcripts (all *F*_3,150_  > 2.70, all *p* < 0.048) except for CR mRNA (*F*_3,150_ = 1.84, *p* = 0.143) (Fig. 1). Relative to UC individuals, PV mRNA levels were significantly lower with large effect sizes in SZ (Cohen's *d*:−1.53; [95% CI: −2.03 to −1.03]) and BD (−1.03; [−1.50, −0.56]) individuals, whereas in MD individuals the moderate effect size (−0.51; [−0.96, −0.06]) did not achieve statistical significance (Fig. 1A). PV mRNA levels in SZ and BD individuals were also significantly lower relative to MD individuals (Fig. 1A). Similarly, levels of KCNS3 mRNA, which is selectively expressed in PV neurons (Georgiev et al., 2012), were significantly lower in SZ individuals than in UC individuals with a large effect size (−1.12; [−1.60, −0.64]) (Fig. 1B). Levels of KCNS3 mRNA in both BD and MD groups were also lower than in the UC group with moderate effect sizes (−0.56; [−1.01, −0.10] and −0.55; [−1.00, −0.09], respectively), but neither difference achieved statistical significance (Fig. 1B). Relative to UC individuals, deficits in SST mRNA levels (Fig. 1C) had large effect sizes in SZ (−0.63; [−1.08, −0.17]) and MD (−0.75; [−1.20, −0.29]) individuals, although the difference fell short of statistical significance in SZ (*p* = 0.064). VIP mRNA levels were significantly lower in SZ relative to UC individuals with a large effect size (−0.78; [−1.24, −0.32]), but did not differ between UC individuals and either BD or MD individuals (Fig. 1D). Finally, GAD67 mRNA levels were significantly lower in SZ relative to UC individuals with a large effect size (−0.85; [−1.31, −0.38]), without a significant difference between UC individuals and either BD or MD individuals (Fig. 1F).

### Patterns of transcript alterations across diagnostic groups

**Figure 2.**
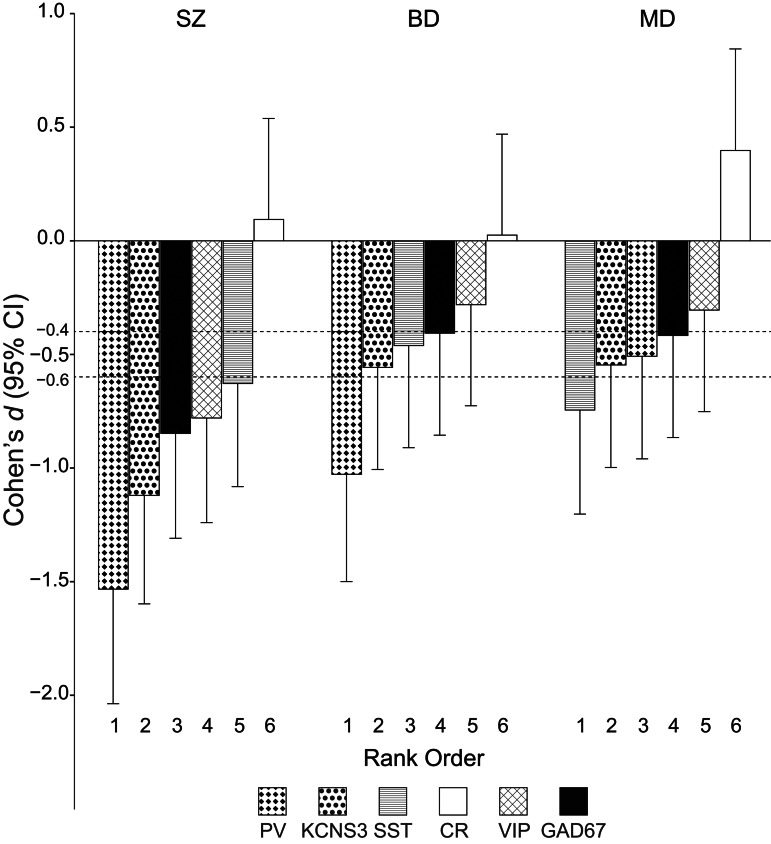
Rank order of transcripts based on Cohen's *d* diagnosis effect size relative to UC individuals for each diagnostic group. Each transcript is represented by columns of different patterns as indicated at the bottom. Choen's *d* levels at −0.4 and −0.6 were indicated by dashed black lines, reflecting the criteria for small, moderate, and large effects defined by |Cohen's *d*| = 0.2–0.4, |Cohen's *d*| = 0.4–0.6, and |Cohen's *d*| ⩾ 0.6, respectively. Vertical lines from each column indicate 95% confidence intervals (CI). PV, parvalbumin; KCNS3, potassium voltage-gated channel modifier subfamily-S member-3; SST, somatostatin; VIP, vasoactive intestinal peptide; CR, calretinin; GAD67, 67 kDa glutamate decarboxylase; SZ, schizophrenia; BD, bipolar disorder; MD, major depression.

To compare the patterns and relative magnitudes of transcript alterations across SZ, BD, and MD individuals, we rank ordered transcripts based on effect sizes (regardless of statistical significance) relative to UC individuals for each diagnostic group (Fig. 2). In the SZ and BD groups, transcripts selective to PV neurons had the largest effect sizes, whereas in the MD group SST mRNA had the largest effect size. Among the five transcripts with an overall significant diagnosis effect, the effect size for each transcript was larger in the SZ group than in either the BD or MD groups, except for SST mRNA which had a slightly larger effect size in the MD group relative to the SZ group.

### Effects of prominent mood symptoms or psychosis on transcript levels within diagnostic groups

**Figure 3.**
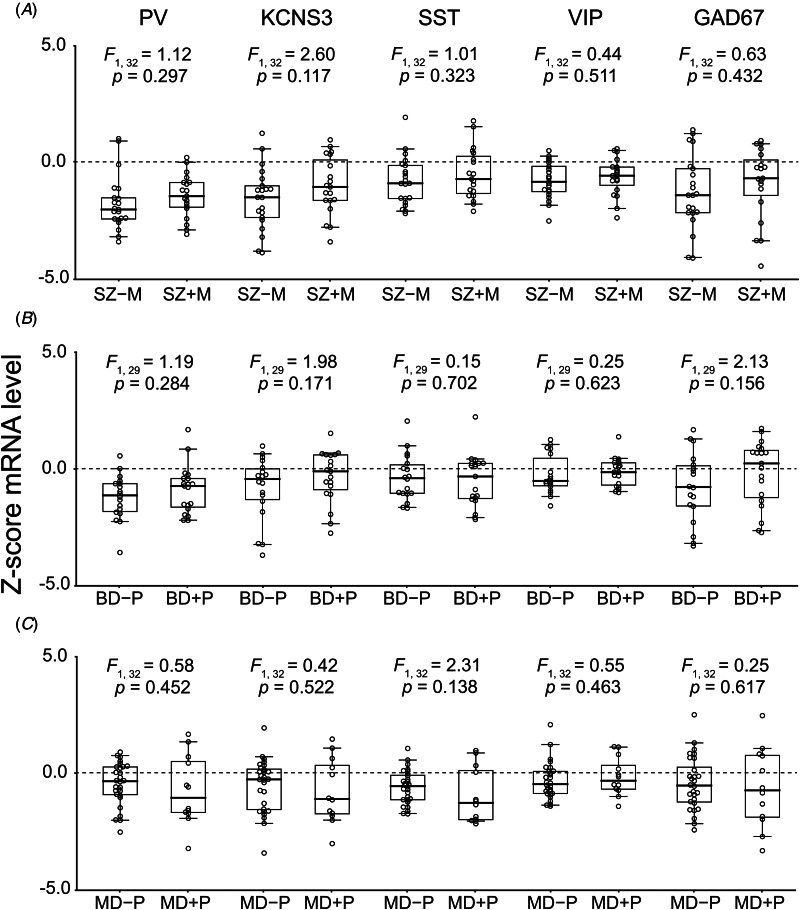
Effects of the history of prominent mood symptoms or psychosis on transcript levels within the schizophrenia (SZ) (A), bipolar disorder (BD) (B), and major depression (MD) (C) groups. Z-scored mRNA levels for parvalbumin (PV), potassium voltage-gated channel modifier subfamily-S member-3 (KCNS3), somatostatin (SST), vasoactive intestinal peptide (VIP), and 67 kDa glutamate decarboxylase (GAD67) relative to the mean and standard deviation of the unaffected comparison (UC) group are compared between subgroups defined by the absence or presence of these clinical features in each diagnostic group. The results of *F*-tests are shown at the top of comparisons between SZ only (SZ − M) (*n* = 21) and schizoaffective disorder (SZ + M) (*n* = 19) (A), between BD without psychosis (BD − P) (*n* = 18) or with psychosis (BD + P) (*n* = 19) (B), and between MD without psychosis (MD − P) (*n* = 28) or with psychosis (MD + P) (*n* = 12) (C). Note that 3 individuals with BD had an unknown lifetime history of psychosis (online Supplementary Table 1). The dashed black line at 0 indicates the mean mRNA levels of the UC group. Circles indicate transcript levels of individuals in corresponding subgroups. Box plots depict the median and 25th and 75th percentiles, with whiskers extending to the 95th percentile of the distribution.

In the SZ individuals, neither PV, KCNS3, SST, VIP, nor GAD67 mRNA levels significantly differed between SZ − M and SZ + M subgroups (all *F*_1,32_ < 2.60, all *p* > 0.117, Fig. 3A), with the mean levels for all transcripts below that of UC individuals in both subgroups. Similarly, none of these mRNA levels differed between BD − P and BD + P subgroups (all *F*_1,29_ < 2.13, all *p* > 0.155, Fig. 3B) or between MD − P and MD + P subgroups (all *F*_1,32_ < 2.31, all *p* > 0.138, Fig. 3C). These comparisons suggest that diagnosis, rather than certain clinical features that cross diagnostic categories, account for the observed patterns of transcript levels.

### Effects of cooccurring factors on transcript levels

**Figure 4.**
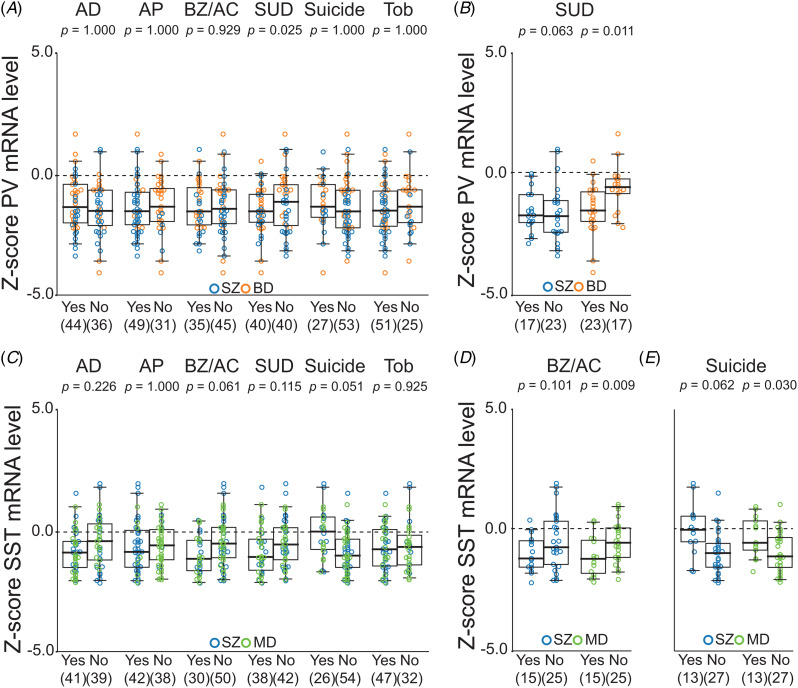
Effects of cooccurring factors, including use of antidepressant (AD), antipsychotic (AP) and benzodiazepine and/or anticonvulsant (BZ/AC) at time of death (ATOD), substance use disorder (SUD) ATOD, death by suicide, and tobacco (Tob) use ATOD, on levels of parvalbumin (PV) and somatostatin (SST) mRNAs. (A) Z-scored PV mRNA levels against the mean and standard deviation of the unaffected comparison (UC) group are compared between individuals with or without each of the cooccurring factors in the combined group of schizophrenia (SZ) and bipolar disorder (BD) individuals. The effect of SUD ATOD, which had a significant effect on PV mRNA levels in the combined group, is further tested separately in the SZ and BD groups (B). (C) Similarly, Z-scored SST mRNA levels are compared between individuals with or without each of the cooccurring factors in the combined group of SZ and major depression (MD) individuals, and the effects of BZ/AC ATOD (D) and suicide (E) are tested separately in the SZ and MD groups. Mean mRNA levels in the UC group are indicated by dashed black lines at 0. Holm-corrected *p*-values from *F*-tests are shown for the main effect of each of six cooccurring factors. Colored circles indicate transcript levels of individuals with SZ (blue), BD (orange), or MD (green). Box plots depict the median, and 25th and 75th percentiles, with whiskers extending to the 95th percentiles of the distribution. Numbers in parentheses below the x-axis indicate the number of individuals with (Yes) or without (No) each corresponding factor.

Because PV mRNA levels were significantly lower in both SZ and BD individuals, we combined these groups to examine the potential influence of cooccurring factors on PV mRNA levels (Fig. 4A). While controlling for the effect of diagnosis, PV mRNA levels did not differ as a function of use of antidepressant, antipsychotic, benzodiazepine/anticonvulsant, or tobacco ATOD, or death by suicide, although substance use disorder ATOD was associated with significantly lower PV mRNA levels (*p* = 0.025) with a small effect size (Cohen's *d*: − 0.29; [95% CI: −0.74 to 0.15]). Further analyses in individual diagnostic groups for PV mRNA (Fig. 4B) revealed that substance use disorder ATOD was associated with significantly lower PV mRNA levels in the BD group (*p* = 0.011) (−0.89; [−1.56, −0.22]). However, relative to UC individuals, mean PV mRNA levels were lower in individuals with or without substance use disorder ATOD for both SZ and BD groups (Fig. 4B).

Because SST mRNA levels were lower in both SZ and MD individuals, we combined these groups to examine the potential influence of cooccurring factors on SST mRNA levels (Fig. 4C). While controlling for the effect of diagnosis, use of antidepressant, antipsychotic, or tobacco ATOD or substance use disorder ATOD did not have a significant effect on SST mRNA levels. However, use of benzodiazepine/anticonvulsant ATOD and death by suicide were associated with trends of lower (*p* = 0.061) (Cohen's *d*: − 0.59; [95% CI: −1.08, −0.17]) and higher (*p* = 0.051) (0.89; [0.43, 1.36]) SST mRNA levels, respectively (Fig. 4C). In the MD group, use of benzodiazepine/anticonvulsant ATOD was associated with significantly lower levels of SST mRNA (*p* = 0.009) (−0.77; [−1.42, −0.11]), although these levels were lower in MD individuals with or without this factor relative to the UC group (Fig. 4D). Suicide was associated with higher SST mRNA levels in both SZ (*p* = 0.062) (1.04; [0.36, 1.71]) and MD (*p* = 0.030) (0.74; [0.08, 1.40]) individuals (Fig. 4E). None of the cooccurring factors showed a significant effect on KCNS3, VIP, or GAD67 mRNA levels in SZ individuals (online Supplementary Figure 1).

We also evaluated a published RNA sequencing data set in the DLPFC of monkeys exposed long-term to vehicle, haloperidol, or clozapine (Fromer et al., 2016). In these data, antipsychotic exposure did not have a significant effect on levels of any of the six transcripts included in the current study (all *p* > 0.130).

Together, these findings suggest that none of the cooccurring factors examined has a major contribution to the observed alterations in transcript levels.

## Discussion

To determine if the presence and magnitude of molecular alterations in GABA neuron subtypes in the DLPFC are conserved or distinct in individuals with SZ, BD, or MD, we systematically compared PV, KCNS3, SST, VIP, CR, and GAD67 mRNAs across these diagnostic groups. Relative to UC individuals, individuals with SZ exhibited deficits with large effect sizes for all transcripts except CR mRNA, whereas individuals with BD exhibited a large effect size deficit only for PV mRNA and individuals with MD only for SST mRNA (Figs 1 and 2). Our large sample size permitted robust assessments of the association of major clinical features with the transcript levels. Neither prominent mood symptoms among individuals with SZ nor psychosis among individuals with BD or MD had a significant effect on levels of any transcripts with significant deficits in these diagnostic groups (Fig. 3). Furthermore, in concert with prior studies (Fromer et al., 2016; Georgiev et al., 2014; Hashimoto et al., 2003; 2008; Morris et al., 2008; Volk, Austin, Pierri, Sampson, & Lewis, 2000), our current assessments of the effects of factors frequently cooccurring with psychiatric diagnoses on transcript levels suggest that none of these factors represent the primary mechanism of the observed alterations in transcript levels (Fig. 4, online Supplementary Figure 1). Together, these findings support the interpretation that different disease processes, rather than certain clinical features or cooccurring factors, account for the distinct patterns of transcript alterations across diagnostic groups.

The magnitude of the diagnosis effect on PV mRNA was the greatest in SZ, intermediate in BD, and smallest in MD, consistent with prior findings for PV mRNA in the same cohort (Chung et al., 2018). Furthermore, levels of GAD67 and KCNS3 mRNAs, which were previously reported to be lower in DLPFC PV neurons in SZ individuals (Georgiev et al., 2014; Hashimoto et al., 2003), were significantly lower in SZ, but not in BD or MD individuals. These findings indicate that PV neurons are most severely affected in SZ, moderately so in BD, and relatively spared, if not intact, in MD individuals. PV neurons, through their divergent perisomatic innervation of pyramidal neurons, regulate the firing of large numbers of nearby pyramidal neurons via GABA release and have essential roles in the generation of gamma oscillations (Tremblay, Lee, & Rudy, 2016), which promote cortical processing for cognitive functions (Cho et al., 2015; Kim, Ahrlund-Richter, Wang, Deisseroth, & Carlen, 2016b; Roux, Wibral, Mohr, Singer, & Uhlhaas, 2012). KCNS3, which encodes a PV neuron-selective subunit of voltage-gated potassium channels (Georgiev et al., 2012), contributes to the regular firing pattern of PV neurons at gamma-band frequencies (Miyamae et al., 2021). Therefore, the combined mRNA deficits for KCNS3 subunit and GAD67, an enzyme for GABA synthesis, in PV neurons are likely to have a substantial effect on the generation of gamma oscillations and may contribute to reports of lower prefrontal gamma-band power during working memory tasks in individuals with SZ (Chen et al., 2014; Cho, Konecky, & Carter, 2006; Minzenberg, Laird, Thelen, Carter, & Glahn, 2009).

The diagnosis effect on SST mRNA levels was greater in both MD and SZ than in BD, consistent with significantly lower SST mRNA levels in MD but not in BD individuals in a prior study of 19 triads of matched UC, BD, and MD individuals that overlapped with the current cohort (Sibille et al., 2011). In SZ, although we detected a large effect size (Cohen's *d* = − 0.63) for SST mRNA, this was somewhat smaller than the effect size (Cohen's *d* = − 0.97) observed in a previous study of 62 matched pairs of SZ and UC individuals (Volk et al., 2016a), suggesting that biological variance in both SZ and UC individuals and technical variance across studies influence the observed magnitude of the diagnosis effect (Lewis, Dienel, & Chung, 2022). Interestingly, in a recent study that quantified SST mRNA levels selectively in layers 2-superficial 3 and deep layers 6-superficial white matter of the DLPFC (Dienel et al., 2023a), the deficit in SST mRNA levels was greater in SZ than in MD; in concert with the present study, these findings suggest that the deficit in SST mRNA levels in other cortical layers may be greater in MD than in SZ. Among SST neurons, Martinotti cells, which innervate distal dendrites of pyramidal neurons, are present mainly in layers 2/3 and 5/6, whereas almost all SST neurons in layer 4 are non-Martinotti cells (Hostetler, Hu, & Agmon, 2023; Tremblay et al., 2016). In rodents, SST neurons in layer 4 were shown to predominantly innervate local PV neurons and provide disinhibition of pyramidal neurons (Xu, Jeong, Tremblay, & Rudy, 2013). Together, these findings suggest that the disease processes of SZ and MD might preferentially affect different subsets of SST neurons involved in dendritic inhibition or disinhibition, respectively, of pyramidal neurons.

Relative to UC individuals, a significant effect on VIP mRNA was detected only in SZ individuals. The effect size (Cohen's *d* = − 0.78) was equivalent to deficit of 21% in the SZ relative to the UC group, which is comparable to deficits of 9–19% reported in studies of different cohorts conducted by two research groups (Fung et al., 2014; Fung et al., 2010; Tsubomoto et al., 2019). These findings suggest that VIP mRNA levels are likely to be lower, at least moderately, in the DLPFC of SZ individuals. In contrast, levels of CR mRNA, which is expressed in a subset of VIP neurons (He et al., 2016; Ma et al., 2022), did not differ between SZ and UC individuals, consistent with multiple prior studies (Chung et al., 2016; Fung et al., 2010; Guillozet-Bongaarts et al., 2014; Hashimoto et al., 2003; Tsubomoto et al., 2019). Given that VIP neurons expressing CR represent a largely different population from VIP neurons that express cholecystokinin (CCK) (He et al., 2016; Tremblay et al., 2016) and that CCK mRNA levels were found to be lower in the DLPFC of SZ individuals (Fung et al., 2010; Hashimoto et al., 2008; Virgo et al., 1995), the current findings suggest that the affected VIP neurons in SZ are those that express CCK. Further studies are needed to test this idea, and to determine if markers of GABA neurotransmission, such as GAD67, are altered in the affected subset of VIP neurons. Such studies will inform the functional impact on DLPFC circuitry of alterations of VIP neurons given that these neurons innervate PV and SST neurons, and thereby provide disinhibition of pyramidal neurons (Tremblay et al., 2016).

Our direct comparisons of GABA neuron subtype-selective marker transcripts and GAD67 mRNA in total gray matter across SZ, BD, and MD suggest that different patterns of molecular alterations in different subtypes of GABA neurons contribute to DLPFC circuitry dysfunction in each disease (Fig. 2). In SZ, transcripts selectively expressed in PV, SST, or VIP neurons, as well as GAD67 mRNA, were all lower with large effect sizes relative to UC individuals. Furthermore, SZ had larger effects than BD or MD on all transcripts except for SST mRNA, which showed a slightly larger deficit in MD than in SZ. Consistent with these findings, prior studies focusing on layers 2-superficial 3 found greater deficits in transcripts for both enzymes of GABA synthesis, GAD65 and GAD67, and for SST mRNA, in SZ than in BD or MD (Dienel et al., 2023a; Dowling, Dienel, Barile, Bazmi, & Lewis, 2023). Together, these findings suggest that SZ is associated with a broader and more severe disturbance of GABA neurons, which might contribute to the more profound cognitive deficits in SZ than in BD and MD (Ceylan et al., 2020; Daban et al., 2006; Reichenberg et al., 2009).

In contrast to SZ, in BD only the deficit in PV mRNA had a large effect size, although the rank order of transcripts based on the magnitude of alterations was similar to SZ (Fig. 2). These findings are consistent with a previous RNA sequencing study which reported that SZ and BD shared multiple gene expression alterations in DLPFC gray matter, although the magnitudes of alterations were larger in SZ than in BD (Enwright & Lewis, 2021). The greater magnitude of GABA neuron transcript deficits in SZ relative to BD in the present study is also consistent with the prior findings that a larger proportion of SZ than of BD individuals cluster separately from UC individuals based on a composite score of four GABA neuron transcript levels in DLPFC gray matter (Volk, Sampson, Zhang, Edelson, & Lewis, 2016b).

Finally, in the present study MD individuals had a large effect size deficit, comparable to that in SZ, only for SST mRNA. However, as described above, a recent study of SST mRNA levels in specific cortical layers suggested that different subsets of SST neurons might be preferentially altered in MD *v.* SZ (Dienel et al., 2023a).

Indeed, data from the current and previous studies together suggest that both shared and distinct disease processes might contribute to differential alterations of GABA neuron subtypes in SZ, BD, and MD. The similar rank orders of transcripts based on the disease effect sizes in SZ and BD (Fig. 2) might reflect the presence of shared genetic (Gratten, Wray, Keller, & Visscher, 2014) and/or environmental (Brown, 2011; Shintani et al., 2023) mechanisms, although the differences in magnitude of the effect sizes suggest that such mechanisms have a greater impact on SZ than on BP. On the other hand, the prominent alteration of SST mRNA in MD suggests the existence of a distinct process that primarily affects certain SST neurons in MD.

## Supporting information

Okuda et al. supplementary material 1 Okuda et al. supplementary material

Okuda et al. supplementary material 2 Okuda et al. supplementary material

## Supplementary material

The supplementary material for this article can be found at https://doi.org/10.1017/S0033291724002344.
