# Supplementary material for: Alterations in inhibitory neuron subtype-selective transcripts in the prefrontal cortex: comparisons across schizophrenia and mood disorders
Source: Psychol Med. 2024 Oct 31;54(14):3896–905. doi: 10.1017/S0033291724002344 (PMC11578916; doi:10.1017/S0033291724002344)
Supplement: Okuda et al. supplementary material 1 — Okuda et al. supplementary material [file S0033291724002344sup001.docx]

Supplementary Figure 1


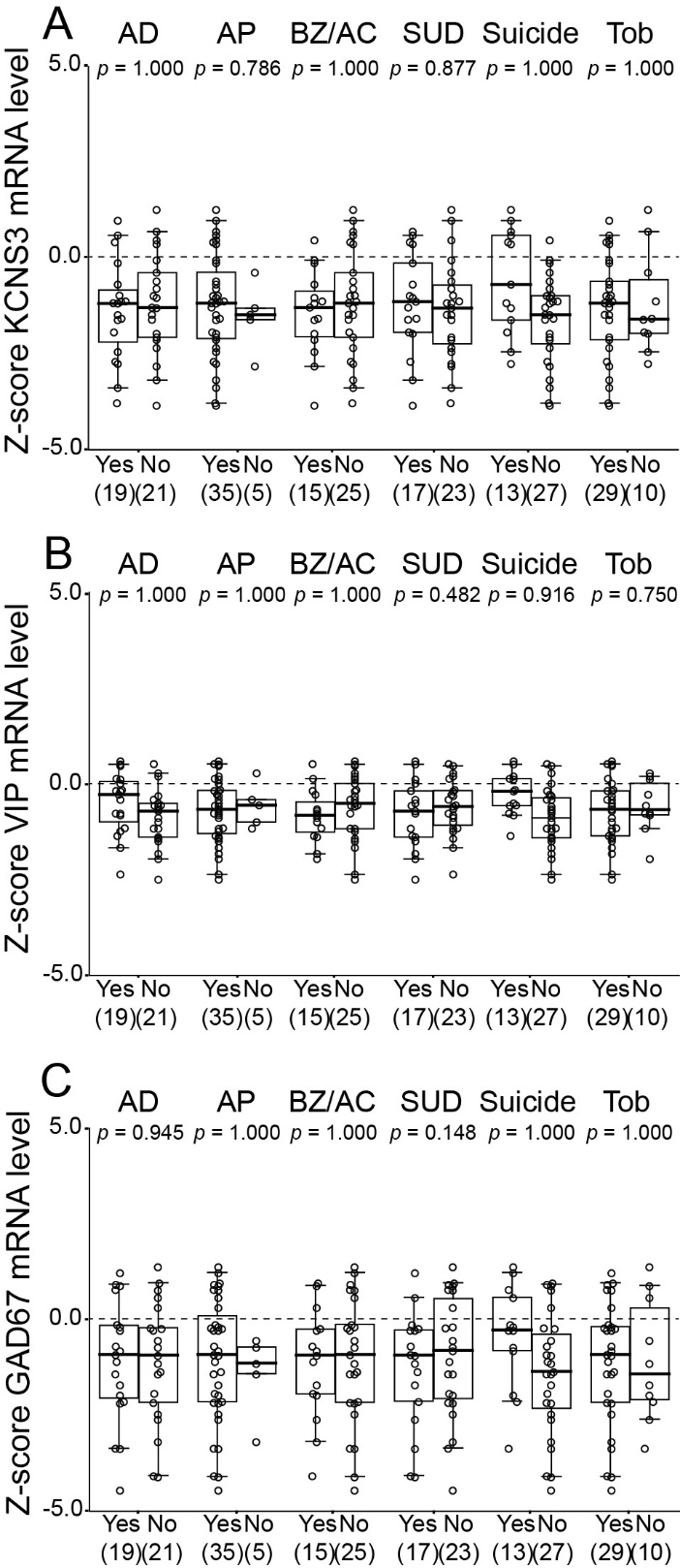
Effects of cooccurring factors, including use of antidepressant (AD), antipsychotic (AP) and benzodiazepine and/or anticonvulsant (BZ/AC) at time of death (ATOD), substance use disorder (SUD) ATOD, death by suicide and tobacco (Tob) use ATOD, on levels of KCNS3 (A), VIP (B) and GAD67 (C) mRNAs in individuals with schizophrenia (SZ).

Z-scored transcript levels against the mean and standard deviation of the unaffected comparison (UC) group are compared between individuals with or without these factors. Mean mRNA levels in the UC group are indicated by dashed black lines at 0. Holm-corrected *p*-values from *F*-tests are shown for the main effect of each of 6 cooccurring factors. Circles indicate transcript levels of individuals with SZ. Box plots depict the median, and 25^th^ and 75^th^ percentiles, with whiskers extending to the 95^th^ percentiles of the distribution. Numbers in parentheses below the x-axis indicate the number of individuals with (Yes) or without (No) each corresponding factor.
